# Systemic inflammation contributes to impairment of quality of life in chronic pancreatitis

**DOI:** 10.1038/s41598-019-43846-8

**Published:** 2019-05-13

**Authors:** Stuart M. Robinson, Sebastian Rasch, Sebastian Beer, Irena Valantiene, Artautas Mickevicius, Elisabeth Schlaipfer, Jelena Mann, Patrick Maisonneuve, Richard M. Charnley, Jonas Rosendahl

**Affiliations:** 1 HPB Unit, Department of Surgery, Newcastle upon Tyne Hospitals NHS Foundation Trust, Newcastle upon Tyne, UK; 2 Fibrosis Research Group, Institute of Cellular Medicine, Newcastle University, Newcastle upon Tyne, UK; 3 Klinik und Poliklinik für Innere Medizin II, Klinikum rechts der Isar, Technische Universität München, Ismaninger Straße 22, 81675 München, Germany; 4 Department for Internal Medicine, Neurology and Dermatology, Division of Gastroenterology, University of Leipzig, Leipzig, Germany; 5 Department of Gastroenterology and Institute for Digestive Research, Lithuanian University of Health Sciences, Kaunas, Lithuania; 6 Centre of Hepatology, Gastroenterology and Dietetics, Vilnius University Hospital Santaros Klinikos & Vilnius University Faculty of Medicine, Vilnius, Lithuania; 7 Division of Epidemiology and Biostatistics, IEO, European Institute of Oncology IRCCS, Milan, Italy; 8 Department of Internal Medicine I, Martin Luther University, Halle, Saale Germany

**Keywords:** Chronic pancreatitis, Pancreas

## Abstract

Chronic pancreatitis (CP) is a fibrotic disorder of the pancreas leading to clinical sequelae like pain and an excess of comorbidity including cardiovascular disease and cancers. The aim of this study was to determine the relationship between systemic inflammation and quality of life in patients with CP. Patients were prospectively recruited and underwent a quality of life assessment (EORTC QLQ-C30 and PAN 28). The serum inflammatory profile was assessed using an MSD 30-plex array. The relationship between clinical variables, inflammatory cytokines and quality of life was determined by a GLM-MANOVA and the individual impact of significant variables evaluated by a second ANOVA. In total, 211 patients with a median age of 53 years were recruited across 5 European centres. Gender, age, nicotine and alcohol abuse were clinical variables associated with altered quality of life. Systemic inflammation with high levels of pro-inflammatory cytokines (Eotaxin, IL-1β, IL-7, IL-8, IL-12/IL-23p40, IL-12p70, IL-13, IL-16, IP-10, MCP-1, MCP-4, MDC, MIP-1a, TARC, TNFß) was associated with diminished quality of life in general and specific domains including pain, physical and cognitive functioning. As conclusion, CP is associated with a systemic inflammatory response that has a negative impact on quality of life and accelerates aging.

## Introduction

Chronic pancreatitis (CP) is a disease characterised by chronic inflammation in the pancreas which ultimately results in organ fibrosis. This process manifests itself clinically in a variety of ways including chronic abdominal pain, steatorrhoea, diabetes mellitus, obstructive jaundice and malabsorption of nutrients leading to secondary conditions such as osteoporosis^1–3^. Unsurprisingly CP is associated with diminished quality of life and a high socioeconomic burden not only to individuals but also to society as a whole^3–5^.

Medical co-morbidity is commonplace in patients with CP. In a retrospective registry study in Denmark Bang *et al*. compared the outcome of 11,972 CP patients with 119,720 age and gender matched controls over a 15-year period^6^. They demonstrated that there was a marked excess mortality in patients with CP over the study period with a mean age of death in patients of 63.7 years as compared to 72.1 years in controls (p < 0.0001). There was an excess of comorbidity in the CP group (78% vs. 38%; p < 0.0001) with diseases including cerebrovascular disease, COPD, ulcer disease, diabetes mellitus and chronic renal disease. Further there was an increased risk of malignancy in the CP group (13.6% vs. 7.9%; p < 0.0001)^6^.

These findings fit with the concept that CP is associated with accelerated biological ageing a process typically characterized by the development of frailty, sarcopenia and diseases associated with older age such as cardiovascular disease and cancers^7^. Specifically there is a recognized increase in the incidence of pancreatic ductal adenocarcinoma in patients with CP, especially in the hereditary form of the disease, which is attributed predominantly to the local inflammatory process acting as an oncogenic driver^8,9^.

While shared risk factors and co-morbidities like smoking and malnutrition contribute to accelerated aging in patients with CP, there is also a strong and established association between accelerated ageing and systemic inflammation – the so-called inflammageing phenotype. Indeed an elevation in markers of systemic inflammation e.g. serum IL-6 and CRP occurs during the normal ageing process despite the absence of infection or other pathophysiological processes to account for their rise^10,11^. In a recent systematic review we have demonstrated that CP is associated with elevated systemic levels of inflammatory mediators such as IL-6, TNFα, IL-8 and members of the IL-1 family^12^. The aim of the current study was to determine the impact of this systemic inflammation on quality of life in patients with CP and thereby determine if inflammageing may be a concept worthy of further exploration in this disease.

## Materials and Methods

The study was approved by the ethical review board of Technische Universität München (Project 104/15) on 02/04/2015 and by each of the relevant ethics authorities in the participating centres. The study protocol conforms to the ethical guidelines of the 1975 Declaration of Helsinki. Written, informed consent was obtained from each patient included in the study.

### Patients

Patients presenting with an established diagnosis of CP attending for review at each of the participating centres were invited to participate in this study. The presence of CP was defined as per the HaPanEU guidelines i.e. either a pain history typical for the disease or, in the absence of pain, the presence of biochemically proven pancreatic exocrine insufficiency. In addition all patients were required to have radiological evidence of CP and the initial diagnosis had to be at least 2 years prior to recruitment^2^.

The exclusion criteria for this study were as follows: an active diagnosis of neoplasia or a disease free interval of less than five years; patients unable to provide informed consent to participate in the study including those less than 18 years of age; patients with Childs-Pugh score B/C liver cirrhosis; patients who were immunosuppressed either as a result of disease (e.g. HIV) or active medical treatment (patients with NSAID as pain medication were not excluded); patients treated with antibiotic therapy within the last month or with an active infective process; patients who had received systemic steroid treatment within the last month; patients with chronic kidney disease stage 4 or greater (eGFR < 30 ml/min) and those who had received surgical treatment of any form to any body-site within the last year.

At the point of recruitment the interviewing doctor completed a study questionnaire and all patients were asked to complete the EORTC QLQC-30 and PAN-28 questionnaires. Blood samples were taken for full blood count, urea & electrolytes, liver function, CRP and HbA_1_C. A serum sample was stored for later analysis at −80 °C.

### Interpretation of quality of life data

Quality of life was assessed by calculating function or symptom scores for each of the domains assessed by the two questionnaires^4,13^. For both types of score a raw score was first calculated as the mean response to each of the component items constituting the domain. For functional scores the following equation was then utilised:

$${\rm{Functional}}\,{\rm{Score}}=\{1-({\rm{Raw}}\,{\rm{Score}}-1)/{\rm{range}}\}\times 100$$

For functional domains a low score implies poorer function and therefore a lower quality of life. In contrast for symptom domains the following formula was utilised:

$${\rm{Symptom}}\,{\rm{Score}}=\{({\rm{Raw}}\,{\rm{Score}}-1)/{\rm{range}}\}\times 100$$

For symptom domains a high score implies more severe symptoms and therefore a poorer quality of life.

### Assessment of inflammatory response

Using stored serum the concentration of 28 inflammatory mediators (Eotaxin, Eotaxin-3, GM-CSF, IFNγ, IL-10, IL-12/IL-23p40, IL-12p70, IL-13, IL-15, IL-16, IL-17, IL-1α, IL-1β, IL-2, IL-4, IL-6, IL-7, IL-8, IP-10, MCP-1, MCP-4, MDC, MIP-1α, MIP-1β, TARC, TNFα, TNF-β, VEGF-A) were assessed using a multiplex array according to the manufacturers instructions (Meso Scale Diagnostics, Maryland, USA).

To determine the relationship between systemic inflammation and quality of life patients with CP were compared to each other and not to healthy controls. Therefore each of the inflammatory mediators measured was categorised as either ‘high’ or ‘low’ based upon the median serum level.

### Statistical analysis

To determine the relationship between the measured variables and quality of life measures we performed a general linear model multivariate analysis of variance (GLM-MANOVA) separately for the QLQ-C30 and PAN-28 questionnaires. We assessed the impact of each factor using Wilks λ with p < 0.05 being considered significant. To ensure that the associations of measured inflammatory variables were independent of key clinical factors (smoking, diabetes mellitus and current alcohol consumption) the analysis was repeated with these factors as co-variates^14^.

Those factors identified as being associated with quality of life on GLM-MANOVA were then entered into a second ANOVA to investigate the association between each factor and the quality of life scale items individually with post-hoc testing using the Bonferroni correction with a p < 0.05 being considered statistically significant.

Numerical variables are presented as mean (±standard deviation). All statistical analysis was carried out using SPSS v. 23 (IBM Corporation, USA).

Any additional data that is not mentioned in the article is provided as supplementary material.

## Results

Across the five participating centres 296 patient were screened for this study, 51 met one or more of the exclusion criteria, 30 refused to participate, 3 were discharged before inclusion and 1 patient had an unsure diagnosis of CP. Altogether a total of 211 patients were recruited for this study. Patient characteristics are summarised in Table 1.

**Table 1:** Summary of patient characteristics.

Participant Age	53 (19–84) years^#^
Gender	156 Male/55 Female
Time since initial diagnosis of CP	7 (2–53) years ^#^
Pancreatitis Aetiology Alcohol Other	148 (70.1%) 63 (29.9%)
Current Smoker (n = 210)^*^	108 (51.4%)
Current Drinker (n = 209)^*^	55 (26.3%)
Diabetes Mellitus (n = 210)^*^	89 (42.4%)
Pain medication with NSAID Mild opioids (WHO class 2) Potent opioids (WHO class 3)	77 (36.5%) 59 (28.0%) 51 (24.2%)
Pancreatic surgery	40 (19.0%)

^*^Numbers in parenthesis represent number of patients with complete data for the variable; ^#^Median (range); WHO = world health organisation.

### Quality of life measures

Across the entire study population the mean global quality of life score was 51.54(±25.38). In the QLQ-C30 questionnaire the lowest function scores were reported for role functioning (60.43 ± 35.60), emotional functioning (61.40 ± 35.60) and social functioning (62.51 ± 34.23) and the highest symptom scores were reported for pain (50.65 ± 34.86), insomnia (48.22 ± 37.61) and fatigue (47.91 ± 30.72). For the PAN-28 questionnaires the highest symptom scores were reported for fear for future health (65.34 ± 33.40), bloated abdomen (47.45 ± 37.79) and pancreatic pain (46.90 ± 31.20). Table 2 summarises quality of life scores across each domain.

**Table 2:** Calculated scores of EORTC QLQ-C30 and PAN28 scales.

Scales	Items	Mean (SD)
**QLQ-C30**		
Global quality of life	Q29,30	51.54 (25.38)
Physical functioning	Q1-5	73.33 (24.97)
Role functioning	Q6-7	60.43 (35.60)
Emotional functioning	Q21-24	61.40 (29.35)
Cognitive functioning	Q20,25	72.79 (26.66)
Social functioning	Q26,27	62.51 (34.23)
Fatigue	Q10,12,18	47.91 (30.72)
Nausea/Vomiting	Q14-15	25.09 (31.57)
Pain	Q9,19	50.65 (34.86)
Dyspnea	Q8	24.43 (31.44)
Insomnia	Q11	48.22 (37.61)
Appetite loss	Q13	38.37 (37.08)
Constipation	Q16	20.65 (29.92)
Diarrhea	Q17	22.77 (28.96)
Financial problems	Q28	35.50 (36.47)
**PAN-28**		
Pancreatic pain	Q31,33,35	46.90 (31.20)
Digestive function	Q36,37	45.40 (36.61)
Jaundice	Q44,45	14.17 (20.03)
Altered bowel functioning	Q46,47	30.47 (28.05)
Body image	Q48,51	37.89 (32.48)
Alcohol related guilt	Q49,50	21.15 (28.40)
Satisfaction with health care	Q55,56	38.85 (35.71)
Sexual functioning	Q57,58	38.83 (38.43)
Bloated abdomen	Q32	47.45 (37.79)
Night pain	Q34	37.75 (36.06)
Taste changes	Q38	20.03 (29.99)
Indigestion	Q39	36.32 (36.09)
Flatulence	Q40	42.79 (33.74)
Weight loss	Q41	30.36 (38.67)
Decreased muscle strength	Q42	36.50 (34.83)
Dry mouth	Q43	38.26 (34.76)
Fear for future health	Q53	65.34 (33.40)
Ability to plan ahead	Q54	41.62 (37.39)

Q = question.

### Clinical variables associated with quality of life

Using the GLM-MANOVA we identified that age (p = 0.03), gender (p = 0.0001) and current smoking status (p = 0.002) were all associated with quality of life outcomes in the QLQ-C30 questionnaire (Supplementary Table 1). In the PAN-28 questionnaire gender (p = 0.04), being a current drinker (p = 0.001) and the presence of diabetes mellitus (p = 0.04) were also negatively associated with quality of life outcomes (Supplementary Table 2).

Subsequent ANOVA analysis revealed that being a current smoker impacted negatively on quality of life across a total of 14 domains in the QLQ-C30 quality of life questionnaire. Male gender was associated with poorer cognitive function, increased dyspnoea scores but better digestive function. Older age was associated with increased financial difficulties. In the PAN 28 being a current drinker was associated with a higher feeling of alcohol related guilt but was associated with less night pain, lower symptom scores for decreased muscle strength and a positive impact on body image. The presence of diabetes mellitus was associated with poorer sexual function but less alcohol related guilt (Table 3).

**Table 3:** Impact of patient characteristics on quality of life.

Predictive Variable	Questionnaire	Quality of Life Domain(s)	Mean Score (Male)	Mean Score (Female)	Bonferroni p-value
Gender	QLQ-C30	Cognitive Function	70.1	80.9	0.01
		Dyspnoea	27.4	15.2	0.02
	PAN-28	Digestive Function	60.7	42.3	0.02
			**Mean Score (Yes)**	**Mean Score (No)**	
Older age	QLQ-C30	Financial Difficulties	43.7	27.6	0.002
Current Drinker	PAN-28	Body Image	27.8	40.7	0.02
		Alcohol Related Guilt	28.5	15.3	0.007
		Night Pain	29.9	42.7	0.05
		Decreased Muscle Strength	28.5	41.3	0.04
Smoker	QLQ-C30	Global Quality of Life	47.8	56.1	0.02
		Physical Functioning	67.1	80.3	<0.0001
		Role Functioning	50.9	70.4	<0.0001
		Emotional Functioning	55.7	68.0	0.004
		Cognitive Function	66.5	80.0	<0.0001
		Social Function	53.4	72.6	<0.0001
		Fatigue	55.5	40.2	<0.0001
		Nausea and Vomiting	33.2	16.5	<0.0001
		Pain	59.8	42.3	<0.0001
		Dyspnoea	30.2	18.2	0.007
		Insomnia	59.1	37.9	<0.0001
		Appetite Loss	48.8	27.0	<0.0001
		Constipation	28.9	12.6	<0.0001
		Financial Difficulties	43.0	29.8	0.01
Diabetes mellitus		Sexual Function	31.9	48.1	0.01
		Alcohol Related Guilt	11.8	25.5	0.003

### Systemic inflammation and quality of life

**Figure 1:**
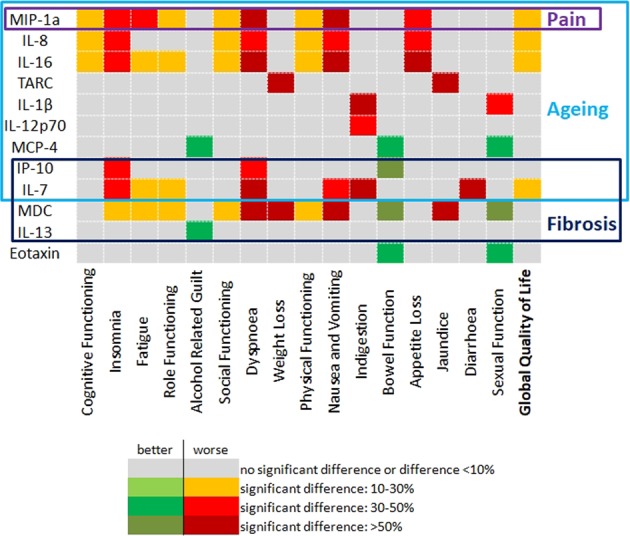
Important aspects of systemic inflammation on quality of life.

Mean levels of inflammatory mediators are presented in Supplementary Table 3. The most relevant associations of inflammatory mediators with quality of life are presented in Table 4 and Fig. 1. Supplementary Table 4 displays all associations with quality of life domains and inflammatory mediators.

**Table 4:** Inflammatory mediators and quality of life.

Predictive Variable	Questionnaire	Quality of Life Domain(s)	Mean Score (Low)	Mean Score (High)	Bonferroni p-value
Eotaxin	PAN-28	Bowel Function	24.5	34.9	0.03
		Abdominal Bloating	54.6	37.6	0.009
IL-7	QLQ-C30	Global Quality of Life	55.8	48.2	0.04
		Fatigue	43.5	53.1	0.03
		Nausea and Vomiting	20.0	29.8	0.03
		Dyspnoea	19.3	30.4	0.01
		Diarrhoea	17.2	27.4	0.01
IL-8	QLQ-C30	Global Quality of Life	56.0	48.3	0.04
		Physical Functioning	78.9	67.8	0.002
		Cognitive Functioning	77.5	68.3	0.02
		Nausea and Vomiting	20.0	29.4	0.04
		Dyspnoea	20.1	29.1	0.05
		Appetite Loss	32.6	44.0	0.03
IL-12/IL-23p40	QLQ-C30	Dyspnoea	29.6	29.5	0.03
IL-16	QLQ-C30	Global Quality of Life	56.7	47.5	0.01
		Physical Functioning	78.4	68.2	0.005
		Cognitive Functioning	77.5	68.1	0.02
		Fatigue	43.0	53.3	0.02
		Nausea and Vomiting	17.2	32.3	0.001
		Dyspnoea	19.2	30.1	0.02
IP-10	QLQ-C30	Dyspnoea	20.2	29.3	0.04
	PAN-28	Bowel Function	19.7	38.3	<0.0001
MCP-4	PAN-28	Bowel Function	23.8	34.8	0.02
MDC	QLQ-C30	Physical Functioning	78.7	67.8	0.003
		Fatigue	43.1	53.3	0.02
		Nausea and Vomiting	17.2	32.4	0.001
		Dyspnoea	17.9	31.5	0.002
		Bowel Function	22.0	36.1	0.002
		Weight Loss	24.2	39.9	0.02
MIP-1a	QLQ-C30	Global Quality of Life	56.5	47.6	0.02
		Physical Functioning	80.2	66.2	<0.0001
		Cognitive Functioning	78.3	67.2	0.004
		Fatigue	40.9	55.6	0.001
		Nausea and Vomiting	17.6	32.1	0.001
		Dyspnoea	18.6	30.8	0.007

Of the measured inflammatory mediators 3 were associated with an impact on quality of life across both QLQ-C30 and PAN-28, these being MDC, IL-7 and IP-10. Across the two questionnaires a high serum MDC level was associated with a negative impact across 12 measured quality of life domains including role functioning, social functioning, fatigue and insomnia. A high serum IL-7 had a negative impact on a total of 10 quality of life domains across the two questionnaires including global quality of life. High IL-7 seemed to be particularly associated with a negative impact on domains related to digestive function including nausea and vomiting, diarrhoea and indigestion. A high serum IP-10 had a negative impact on quality of life across a total of 4 domains.

High expression of a further 5 inflammatory mediators (IL-8, IL-12/IL-23p40, IL-16, MIP1α and TNFβ) were associated with a negative impact on quality of life domains measured by QLQ-C30. Of these high serum levels of IL-8, IL-16 and MIP1α were associated with a negative impact on global quality of life. Individually a high serum MIP1α had a negative impact across 12 measured domains, IL-16 across 10 domains, IL-8 across 8 domains and both IL-12/IL-23p40 and TNFβ on one domain each.

For the PAN-28 questionnaire a high expression of 6 of the measured inflammatory mediators (Eotaxin, IL-12p70, IL-13, IL-1β, MCP1, MCP4, TARC) was associated with a negative impact on quality of life. Whilst a high serum MIP1β was associated with a negative impact on quality of life this association was lost when the GM-MANOVA analysis was adjusted for diabetes mellitus and it was thus not considered further (λ = 0.802; p = 0.07, Supplementary Table 2). A high serum MCP4 had a negative impact on 4 of the measured quality of life domains. Eotaxin, MCP-1 and TARC impacted on 3 domains each; IL-12p70 and IL-1β on 2 domains each and IL-13 on one domain.

## Discussion

It has been reported in a variety of other diseases that elevated serum inflammatory cytokine levels are associated with an impaired quality of life in patients^15–17^. In this study we have shown that there is a strong link between a diminished quality of life and systemic inflammation in patients with CP. Several pro-inflammatory mediators are associated with reduced quality of life in general or with worse specific function and symptom scores of the QLQ-C30 and PAN 28 questionnaires. While some of the inflammatory mediators were described in recent clinical and pre-clinical studies dealing with this issue, changed serum levels of most of the identified inflammatory mediators have not been associated to CP yet^12,18^. Patients with CP suffer from a diminished quality of life in general. Our results indicate three aspects with a particular impact on quality of life: pain as the most prevalent symptom, pro-inflammatory mediators involved in fibrosis as well as in accelerated aging.

Amongst the symptom scores pain stands out with an apparently high score. This finding is in accordance with previous studies and questions the current analgesic treatment of these patients. Analgesic medication alone often does not result in sufficient pain control^13,19^. Otherwise, it has been demonstrated that smoking influences disease progression in chronic pancreatitis as measured by calcifications within the gland and the development of diabetes mellitus^20^. Of the measured clinical variables smoking impacted on 14 individual domains of the QLQ-C30 questionnaire including those such as pain and global quality of life. This confirms the results of recent publications that also showed that pain and nicotine abuse negatively influence quality of life in patients with CP^21,22^. Whether pain and smoking status are independent variables influencing quality of life as described by Machicado *et al*. or nicotine abuse influences quality of life by its impact on pain still has to be evaluated. The fact that smoking cessation might be a potent additive option to treat pain and improve quality of life in those patients highlights the need for targeted support for this patient cohort.

In this study we have shown for the first time, to our knowledge, an association between an elevated MIP-1a level and poorer quality of life. MIP-1a is primarily described as a chemokine attracting macrophages and neutrophils to areas of tissue injury^23^, indeed MIP-1a is thought to play a key role in attracting macrophages to the injured pancreas in murine models of alcohol induced pancreatitis^24^.

Whilst it may be that MIP-1a is simply a surrogate for pancreatic injury and therefore a diminished quality of life the chemokine has been linked with more systemic effects that may also affect quality of life. For example elevated serum levels of MIP-1a have been linked to changes in mood and altered cognition and this would fit with our finding that MIP-1a was significantly associated with poorer cognitive functioning^25,26^. There is also evidence from animal models of sciatic nerve ligation that MIP-1a contributes to the pathogenesis of neuropathic pain although we did not demonstrate an association with pain in the current study^27^.

Fibrosis is one key element in pathogenesis and progression of CP and driven among others by Il-13^28^. Also, an increased serum MDC level was associated with a negative impact on quality of life across both questionnaires. MDC is associated with pulmonary fibrosis and in those patients with idiopathic pulmonary fibrosis, it is associated with poorer outcomes^29,30^. Similarly whilst little is known of the role of IL-7 in CP it does play a fundamental role in the biology of fibroblast activation. Also IP-10 has been identified as a biomarker of chronic liver disease where it promotes inflammation and fibrosis and so may represent a part of the fibrogenic process in CP^31,32^.

Another area that has not received much exploration to date is the impact of systemic inflammation on the development of frailty and ageing. Typically ageing is associated with triggering of the NF-κB, IL-1α, TGF-β and IL-6 pathways^33^. In addition, a senescence associated secretory phenotype has been described and many inflammatory mediators identified in this analysis belong to this secretory phenotype. As a negative regulator of TGF-β signalling, Il-7 is a component of the senescence associated phenotype^34,35^. An elevated serum IP-10 level is also considered to be a biomarker of the ageing process^36^. Of the other inflammatory mediators identified as having an association with quality of life in this study several are known to be associated with senescence and ageing including IL-8, MIP-1a, IL-13, IL-1β and MCP-4^35^. Declines in both physical and cognitive functioning are key hallmarks of the ageing process. Il-8 and 16 as well as MDC and MIP-1a are associated with significantly lower scores for physical and cognitive functioning.

In addition several of the other inflammatory mediators are associated with poorer quality of life and ageing in other diseases. For example an elevated serum IL-16 level is associated with neurocognitive impairment in those living with HIV and an elevated serum IL-12p70 is associated with poorer cognitive function in ageing adults^37,38^.

Whether cellular senescence is the cause of the inflammatory pattern seen in those patients with a poorer quality of life, or, whether it is merely a consequence of ongoing cellular injury within the pancreas remains to be determined although there is evidence that senescence does occur within pancreatic acinar cells and stellate cells in patients with CP^39,40^.

Further studies are needed to evaluate whether the inflammatory mediators are causative for the recorded symptoms and changes in quality of life and whether these inflammatory mediators might then qualify as potential therapeutic targets to treat symptoms and increase quality of life in patients with CP.

## Conclusion

This study has demonstrated that diminished quality of life in CP is associated with elevated serum levels of several inflammatory mediators which cannot be explained by clinical variables such as smoking status and diabetes mellitus. Also inflammatory mediators might be involved in disease progression and accelerated aging in patients with CP. It remains to be determined whether this effect is causal or merely a surrogate for ongoing pancreatic injury but it is worthy of further exploration given the strong association with CP and poor systemic health. In addition, smoking has a strong influence on pain and quality of life in general in those patients.

## Supplementary information

Dataset 1

## Data Availability

All data generated or analysed during this study, that are not included in this published article (and its Supplementary Information Files), are available from the corresponding author on reasonable request.
