# Supplementary material for: Systemic inflammation contributes to impairment of quality of life in chronic pancreatitis
Source: Sci Rep. 2019 May 13;9:7318. doi: 10.1038/s41598-019-43846-8 (PMC6513859; doi:10.1038/s41598-019-43846-8)
Supplement: Supplementary file 1 — Dataset 1 [file 41598_2019_43846_MOESM1_ESM.pdf]

Systemic inflammation contributes to impairment of quality of life in chronic pancreatitis

Authors: Stuart M Robinson[1,2,#], Sebastian Rasch[3,#,*], Sebastian Beer[4], Irena Valantiene[5], Artautas Mickevicius[6], Elisabeth Schlaipfer[3], Jelena Mann[1,2], Patrick Maisonneuve[7], Richard M Charnley[1], Jonas Rosendahl[8]

# These authors contributed equally to this work.

[1] HPB Unit, Department of Surgery, Newcastle upon Tyne Hospitals NHS Foundation Trust, Newcastle upon Tyne, UK

[2] Fibrosis Research Group, Institute of Cellular Medicine, Newcastle University, Newcastle upon Tyne, UK

[3] II. Medizinische Klinik und Poliklinik, Klinikum rechts der Isar, Technische Universität München, Ismaninger Straße 22, 81675 München, Germany

[4] Department for Internal Medicine, Neurology and Dermatology, Division of Gastroenterology, University of Leipzig, Leipzig, Germany

[5] Department of Gastroenterology and Institute for Digestive Research, Lithuanian University of Health Sciences, Kaunas, Lithuania

[6] Centre of Hepatology, Gastroenterology and Dietetics, Vilnius University Hospital Santaros Klinikos & Vilnius University Faculty of Medicine, Vilnius, Lithuania

[7] Division of Epidemiology and Biostatistics, IEO, European Institute of Oncology IRCCS, Milan, Italy

[8] Department of Internal Medicine I, Martin Luther University, Halle (Saale), Germany

Supplementary Table 1: MANOVA test of the overall effect of patients characteristics and measured inflammatory mediators in serum on the EORTC QLQ-C30 scale

	**QLQ-C30**							
	One-factor model		Adjusted Model - Smoking		Adjusted Model – Diabetes Mellitus		Adjusted Model – Current Drinker	
**Variable**	Wilk's λ	P	Wilk's λ	P	Wilk's λ	P	Wilk's λ	P
Age: ≤ 53 vs > 53 years	**0.859**	**0.02**	**0.866**	**0.04**	**0.859**	**0.02**	**0.861**	**0.03**
Gender: female vs. male	**0.779**	**<0.0001**	**0.782**	**<0.0001**	**0.779**	**<0.0001**	**0.779**	**<0.0001**
Pancreatitis etiology: Alcohol vs other	0.883	0.09	0.891	0.14	0.884	0.10	0.882	0.09
Current smoker: Yes vs no	**0.816**	**0.001**	-	-	**0.816**	**0.001**	**0.820**	**0.002**
Current drinker: Yes vs no	0.918	0.41	0.924	0.51	0.920	0.44	-	-
Diabetes Mellitus: Yes vs no	0.914	0.35	0.915	0.38	-	-	0.914	0.36
Eotaxin: High vs Low	0.932	0.67	0.935	0.75	0.925	0.57	0.933	0.70
Eotaxin3: High vs low	0.880	0.10	0.870	0.06	0.886	0.14	0.892	0.18
GM-CSF: High vs low	0.876	0.08	0.876	0.09	0.884	0.12	0.865	0.05
IFNγ: High vs low	0.890	0.16	0.886	0.14	0.892	0.18	0.888	0.15
IL-2: High vs low	0.876	0.08	0.867	0.06	0.876	0.08	0.878	0.10
IL-4: High vs low	0.938	0.74	0.940	0.78	0.937	0.72	0.933	0.69
IL-5: High vs low	0.932	0.65	0.931	0.65	0.932	0.66	0.932	0.67
IL-6: High vs low	0.878	0.09	0.904	0.29	0.880	0.10	0.876	0.09
IL-7: High vs low	**0.855**	**0.03**	**0.855**	**0.03**	**0.853**	**0.02**	**0.845**	**0.02**
IL-8: High vs low	0.866	0.05	0.866	0.05	0.871	0.06	**0.860**	**0.04**
IL-10: High vs low	0.901	0.25	0.902	0.27	0.910	0.36	0.897	0.23
IL-12/IL-23p40: High vs low	**0.851**	**0.02**	**0.836**	**0.01**	**0.848**	**0.02**	**0.849**	**0.02**
IL-12p70	0.913	0.38	0.908	0.34	0.914	0.41	0.912	0.39
IL-13: High vs low	0.956	0.93	0.943	0.81	0.957	0.93	0.957	0.94
IL-15: High vs low	0.875	0.07	0.880	0.10	0.882	0.11	0.872	0.07
IL-16: High vs low	**0.846**	**0.02**	**0.834**	**0.01**	**0.846**	**0.02**	**0.852**	**0.03**
IL-17a: High vs low	0.902	0.23	0.905	0.30	0.902	0.26	0.901	0.26
IL-1α: High vs low	0.947	0.84	0.955	0.92	0.946	0.84	0.950	0.88
IL-1β: High vs low	0.888	0.14	0.878	0.09	0.884	0.12	0.892	0.18
IP10: High vs low	**0.847**	**0.02**	**0.841**	**0.01**	**0.855**	**0.03**	**0.852**	**0.03**
MCP1: High vs low	0.916	0.42	0.919	0.48	0.916	0.43	0.918	0.47
MCP4: High vs low	0.934	0.68	0.935	0.71	0.934	0.69	0.933	0.69
MIP1a: High vs low	**0.825**	**0.004**	**0.791**	**<0.0001**	**0.834**	**0.007**	**0.818**	**0.003**
MIP1b: High vs low	0.884	0.12	0.874	0.08	0.883	0.12	0.893	0.20
MDC: High vs low	**0.841**	**0.01**	**0.847**	**0.02**	**0.844**	**0.01**	**0.844**	**0.02**
TARC: High vs low	0.919	0.47	0.932	0.66	0.916	0.43	0.918	0.47
TNFa: High vs low	0.938	0.74	0.923	0.54	0.942	0.79	0.939	0.77
TNFb: High vs low	**0.856**	**0.03**	**0.860**	**0.04**	**0.854**	**0.03**	**0.857**	**0.03**
VEGF: High vs low	0.905	0.29	0.918	0.46	0.906	0.30	0.908	0.34

Results in bold: $p<0.05$

Supplementary Table 2: MANOVA test of the overall effect of patients characteristics and measured inflammatory mediators in serum on the EORTC PAN-28 scale

	**PAN28**							
	One-factor model		Adjusted Model - Smoking		Adjusted Model – Diabetes Mellitus		Adjusted Model – Current Drinker	
**Variable**	Wilk's λ	P	Wilk's λ	P	Wilk's λ	P	Wilk's λ	P
Age: ≤ 53 vs > 53 years	0.895	0.70	0.893	0.69	0.897	0.72	0.892	0.68
Gender: female vs. male	**0.795**	**0.04**	0.799	0.05	**0.792**	**0.04**	**0.791**	**0.04**
Pancreatitis etiology: Alcohol vs other	0.868	0.42	0.897	0.72	0.860	0.35	0.874	0.50
Current smoker: Yes vs no	0.840	0.20			0.833	0.16	0.831	0.16
Current drinker: Yes vs no	**0.717**	**0.001**	**0.712**	**0.001**	**0.716**	**0.001**		
Diabetes Mellitus: Yes vs no	0.800	0.05	**0.793**	**0.04**			**0.794**	**0.04**
Eotaxin: High vs Low	**0.708**	**0.001**	**0.709**	**0.001**	**0.714**	**0.002**	**0.712**	**0.001**
Eotaxin3: High vs low	0.829	0.17	0.829	0.18	0.832	0.20	0.845	0.29
GM-CSF: High vs low	0.851	0.32	0.852	0.34	0.842	0.26	0.849	0.32
IFNγ: High vs low	0.905	0.81	0.899	0.77	0.904	0.82	0.907	0.84
IL-2: High vs low	0.850	0.31	0.841	0.25	0.841	0.25	0.843	0.27
IL-4: High vs low	0.901	0.78	0.902	0.80	0.899	0.77	0.901	0.80
IL-5: High vs low	0.880	0.58	0.876	0.53	0.877	0.56	0.880	0.60
IL-6: High vs low	0.819	0.12	0.822	0.14	0.825	0.15	0.838	0.24
IL-7: High vs low	**0.790**	**0.04**	**0.788**	**0.04**	**0.786**	**0.04**	**0.768**	**0.02**
IL-8: High vs low	0.806	0.08	0.803	0.07	0.807	0.09	0.809	0.10
IL-10: High vs low	0.857	0.37	0.860	0.40	0.870	0.50	0.867	0.47
IL-12/IL-23p40: High vs low	0.854	0.34	0.863	0.44	0.853	0.34	0.856	0.38
IL-12p70	**0.773**	**0.02**	**0.772**	**0.02**	**0.779**	**0.03**	**0.776**	**0.03**
IL-13: High vs low	**0.777**	**0.02**	**0.782**	**0.03**	**0.777**	**0.03**	**0.780**	**0.03**
IL-15: High vs low	0.799	0.06	0.802	0.07	0.795	0.06	0.815	0.12
IL-16: High vs low	0.860	0.39	0.854	0.35	0.855	0.36	0.858	0.40
IL-17a: High vs low	0.930	0.96	0.929	0.96	0.929	0.96	0.924	0.94
IL-1α: High vs low	0.878	0.57	0.861	0.41	0.878	0.58	0.879	0.60
IL-1β: High vs low	**0.712**	**0.001**	**0.713**	**0.001**	**0.731**	**0.003**	**0.712**	**0.001**
IP10: High vs low	**0.788**	**0.04**	**0.787**	**0.04**	0.798	0.06	0.792	0.05
MCP1: High vs low	**0.722**	**0.002**	**0.724**	**0.002**	**0.722**	**0.002**	**0.733**	**0.003**
MCP4: High vs low	**0.724**	**0.002**	**0.726**	**0.002**	**0.746**	**0.006**	**0.722**	**0.002**
MIP1a: High vs low	0.801	0.06	0.798	0.06	0.826	0.16	0.806	0.09
MIP1b: High vs low	0.794	0.05	0.792	0.05	0.802	0.07	0.792	0.05
MDC: High vs low	**0.693**	**<0.0001**	**0.698**	**<0.0001**	**0.690**	**<0.0001**	**0.688**	**<0.0001**
TARC: High vs low	**0.703**	**0.001**	**0.707**	**0.001**	**0.695**	**<0.0001**	**0.698**	**<0.0001**
TNFa: High vs low	0.846	0.28	0.850	0.32	0.861	0.41	0.852	0.34
TNFb: High vs low	0.903	0.80	0.899	0.77	0.900	0.79	0.905	0.83
VEGF: High vs low	0.905	0.81	0.909	0.85	0.901	0.79	0.908	0.85

Results in bold: $p<0.05$

Supplementary Table 3: Median serum levels of inflammatory mediators in pg/ml

Cytokine	Median	± SD
Eotaxin	130.6	100.8
Eotaxin_3	14.1	312.6
GM_CSF	0.10	0.25
IFN_g	4.78	40.72
IL_10	0.31	0.80
IL_12	86.19	67.18
IL_12p70	0.11	0.51
IL_13	0.27	2.06
IL_15	2.64	0.92
IL_16	231.32	194.22
IL_17A	3.65	6.40
IL_1a	0.00	2.01
IL_1b	0.00	1.84
IL_2	0.11	1.11
IL_4	0.02	0.10
IL_5	0.00	0.77
IL_6	0.98	15.54
IL_7	14.24	12.80
IL_8	16.83	238.5
IP_10	96.50	219.73
MCP_1	160.70	92.64
MCP_4	77.15	61.83
MDC	740.88	559.89
MIP_1a	11.75	231.67
MIP_1b	65.54	357.11
TARC	148.07	226.03
TNFa	2.96	8.72
TNFb	0.18	0.55
VEGF	142.28	151.18

Supplementary Table 4: All associations between inflammatory mediators and quality of life

Predictive Variable	Questionnaire	Quality of Life Domain(s)	Mean Score (Low)	Mean Score (High)	Bonferroni p-value
Eotaxin	PAN-28	Bowel Function	24,5	34,9	0,029
		Sexual Function	30,8	47,3	0,011
		Abdominal Bloating	54,6	37,6	0,009
IL-1β	PAN-28	Sexual Function	44,5	31,8	0,048
		Indigestion	31	48,4	0,009
IL-7	QLQ-C30	Global Quality of Life	55,8	48,2	0,038
		Role Functioning	65,6	54,3	0,029
		Fatigue	43,5	53,1	0,03
		Nausea and Vomiting	20	29,8	0,032
		Dyspnoea	19,3	30,4	0,014
		Insomnia	43,2	54,4	0,039
		Diarrhoea	17,2	27,4	0,014
	PAN-28	Healthcare satisfaction	31,1	43,1	0,044
		Indigestion	29,9	48,3	0,005
		Ability to plan ahead	33,8	48,8	0,017
IL-8	QLQ-C30	Global Quality of Life	56	48,3	0,038
		Physical Functioning	78,9	67,8	0,002
		Cognitive Functioning	77,5	68,3	0,019
		Social Functioning	68,1	58,2	0,045
		Nausea and Vomiting	20	29,4	0,038
		Dyspnoea	20,1	29,1	0,047
		Insomnia	41,4	55,7	0,009
		Appetite Loss	32,6	44	0,034
IL-12/IL-23p40	QLQ-C30	Dyspnoea	29,6	29,5	0,029
IL-12p70	PAN-28	Indigestion	32	47,4	0,02
		Fear for future health	60,7	76,6	0,003
IL-13	PAN-28	Alcohol Related Guilt	26,3	13,7	0,008
IL-16	QLQ-C30	Global Quality of Life	56,7	47,5	0,012
		Physical Functioning	78,4	68,2	0,005
		Role Functioning	66	54,3	0,025
		Cognitive Functioning	77,5	68,1	0,016
		Social Functioning	68,1	58,1	0,043
		Fatigue	43	53,3	0,019
		Nausea and Vomiting	17,2	32,3	0,001
		Dyspnoea	19,2	30,1	0,015
		Insomnia	41,3	55,9	0,007
		Appetite Loss	29	47,7	<0,0001
IP-10	QLQ-C30	Dyspnoea	20,2	29,3	0,043
		Insomnia	41,1	56,4	0,005
	PAN-28	Bowel Function	19,7	38,3	<0,0001
		Flatulence	33,8	45,5	0,041
MCP-1	PAN-28	Jaundice	10,7	19	0,02
		Healthcare Satisfaction	46,9	28,7	0,002
		Abdominal Bloating	55,7	38,8	0,009
MCP-4	PAN-28	Bowel Function	23,8	34,8	0,019
		Alcohol Related Guilt	26,7	13,5	0,005
		Sexual Function	31,9	45,2	0,039
		Ability to plan ahead	49,6	33,3	0,009
MDC	QLQ-C30	Physical Functioning	78,7	67,8	0,003
		Role Functioning	66,1	54	0,019
		Social Functioning	71,7	54,4	<0,0001
		Fatigue	43,1	53,3	0,021
		Nausea and Vomiting	17,2	32,4	0,001
		Dyspnoea	17,9	31,5	0,002
		Insomnia	42,7	54,7	0,027
		Financial Difficulties	30,1	42	0,026
	PAN-28	Jaundice	9,6	20,2	0,003
		Bowel Function	22	36,1	0,002
		Sexual Function	29,3	47,2	0,005
		Weight Loss	24,2	39,9	0,019
MIP-1a	QLQ-C30	Global Quality of Life	56,5	47,6	0,017
		Physical Functioning	80,2	66,2	<0,0001
		Role Functioning	67	53,1	0,007
		Cognitive Functioning	78,3	67,2	0,004
		Social Functioning	72,6	53,5	<0,0001

		Fatigue	40,9	55,6	0,001
		Nausea and Vomiting	17,6	32,1	0,001
		Dyspnoea	18,6	30,8	0,007
		Insomnia	40,5	56,9	0,003
		Appetite Loss	33	43,8	0,043
		Constipation	15,4	26,1	0,016
		Financial Difficulties	27,6	44,6	0,001
TARC	PAN-28	Jaundice	10,1	19,4	0,009
		Body Image	30,7	42,1	0,036
		Weight Loss	24,3	39,2	0,026
TNFb	QLQ-C30	Financial Difficulties	42,4	29,2	0,014
